# Supplementary material for: The role of connectivity on malaria dynamics across areas with contrasting control coverage in the Peruvian Amazon
Source: PLoS Negl Trop Dis. 2024 Nov 4;18(11):e0012560. doi: 10.1371/journal.pntd.0012560 (PMC11534198; doi:10.1371/journal.pntd.0012560)
Supplement: S1 Methods — (DOCX) [file pntd.0012560.s001.docx]

**Supplementary Methods 1: Network Analysis – Data cleaning**

The origin-destination data sets contained duplicates because the calculation of distance and travel time was performed against the entire set. In other words, there could be a connection from village A to B and from B to A, which gave the same distance and travel time. We removed the duplicates and also filtered out those connections that had a calculation equal to zero (connection between the same community). Overall, 1’671,706 distance connections and 1’627,314 travel time connections were computed. Complete case dataset was constructed resulting in a single origin-destination dataset with 1’627,314 connections, having one column for distance and another one for travel time.

The average annual forest loss for each village from 2009 to 2018 was then calculated. Those villages with an average annual forest loss of zero and a population of zero were then excluded from further processing. From the total number of villages and connections, 1634 villages and 73,946 connections remained for analysis. After this, we grouped the connections by watershed and filtered out those that had only one connection, leaving us with 31 watersheds, 73944 connections and 1608 communities (Supplementary Figure 1).
