# Supplementary material for: The role of connectivity on malaria dynamics across areas with contrasting control coverage in the Peruvian Amazon
Source: PLoS Negl Trop Dis. 2024 Nov 4;18(11):e0012560. doi: 10.1371/journal.pntd.0012560 (PMC11534198; doi:10.1371/journal.pntd.0012560)
Supplement: S2 Methods — (DOCX) [file pntd.0012560.s002.docx]

**Supplementary Methods 2: Description of centrality metrics**

The strength centrality of a particular node is the sum of the weights of all its adjacent edges [1]. The weights here are interpreted as measures of attraction between nodes. On the other hand, the closeness centrality measures how “close” is a node to other nodes it is connected and is defined as the inverse sum of the weights of the adjacent edges of a particular node, given that the weights are measuring the farness between the nodes [1]. Using this same interpretation of the weights, if for every pair of nodes in the graph we find the path or sequence of edges that connects the nodes that minimize the sum of the edge weights, that is, the shortest path, then the between centrality of a certain node is the proportion of shortest paths that passes through this node [1]. Finally, the eigenvector centrality uses the interpretation of weights as connection strength to give higher scores to the nodes that are more connected to other nodes with high scores [1]. In a social network setting, for example, a person with high eigenvector score is a popular one that has relatively more popular friends. Mathematically, the scores are calculated as the eigenvector components of the weighted adjacency matrix of the graph.

**References**

1. Kolaczyk ED, Csárdi G. Statistical Models for Network Graphs. In: Kolaczyk ED, Csárdi G, editors. Statistical Analysis of Network Data with R. New York, NY: Springer; 2014. pp. 85–109. doi:10.1007/978-1-4939-0983-4_6
