# Supplementary figures and images for: The role of connectivity on malaria dynamics across areas with contrasting control coverage in the Peruvian Amazon
Source: PLoS Negl Trop Dis. 2024 Nov 4;18(11):e0012560. doi: 10.1371/journal.pntd.0012560 (PMC11534198; doi:10.1371/journal.pntd.0012560)

**Supplementary Figure 1. Data flowchart of the analytical dataset.**

**
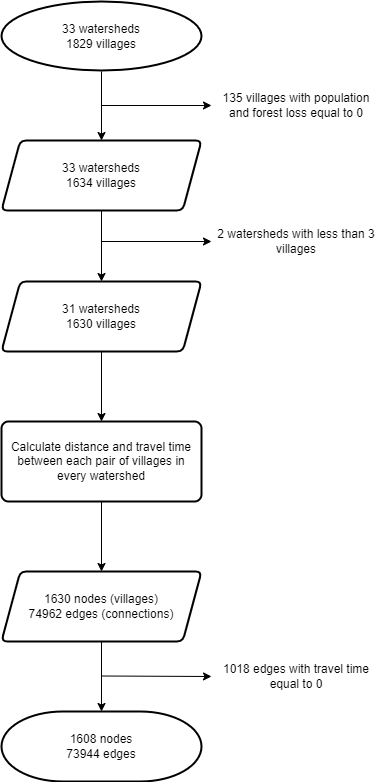
**

Supplement: S1 Fig — (DOCX) [file pntd.0012560.s004.docx]
