# Supplementary material for: The role of connectivity on malaria dynamics across areas with contrasting control coverage in the Peruvian Amazon
Source: PLoS Negl Trop Dis. 2024 Nov 4;18(11):e0012560. doi: 10.1371/journal.pntd.0012560 (PMC11534198; doi:10.1371/journal.pntd.0012560)
Supplement: S3 Fig — (DOCX) [file pntd.0012560.s006.docx]

**Supplementary Figure 3. Distribution of distance and travel time between dyad villages in each watershed in the Loreto department in the Peruvian Amazon.**

**
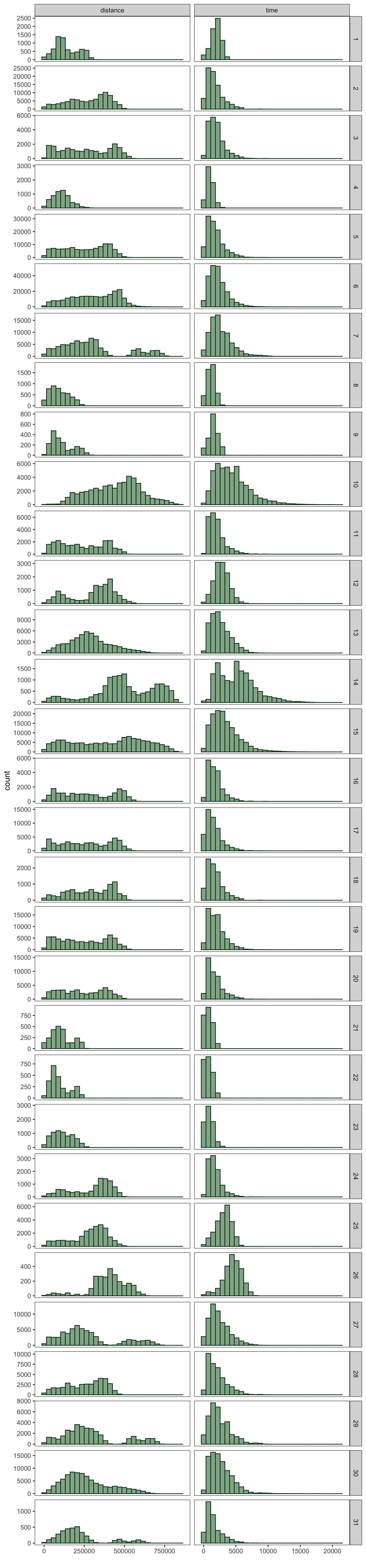
**
