# Supplementary material for: The role of connectivity on malaria dynamics across areas with contrasting control coverage in the Peruvian Amazon
Source: PLoS Negl Trop Dis. 2024 Nov 4;18(11):e0012560. doi: 10.1371/journal.pntd.0012560 (PMC11534198; doi:10.1371/journal.pntd.0012560)
Supplement: S4 Fig — (DOCX) [file pntd.0012560.s007.docx]

**Supplementary Figure 4. Consensus graph of the network of villages by watersheds in the Loreto department in the Peruvian Amazon.**

**
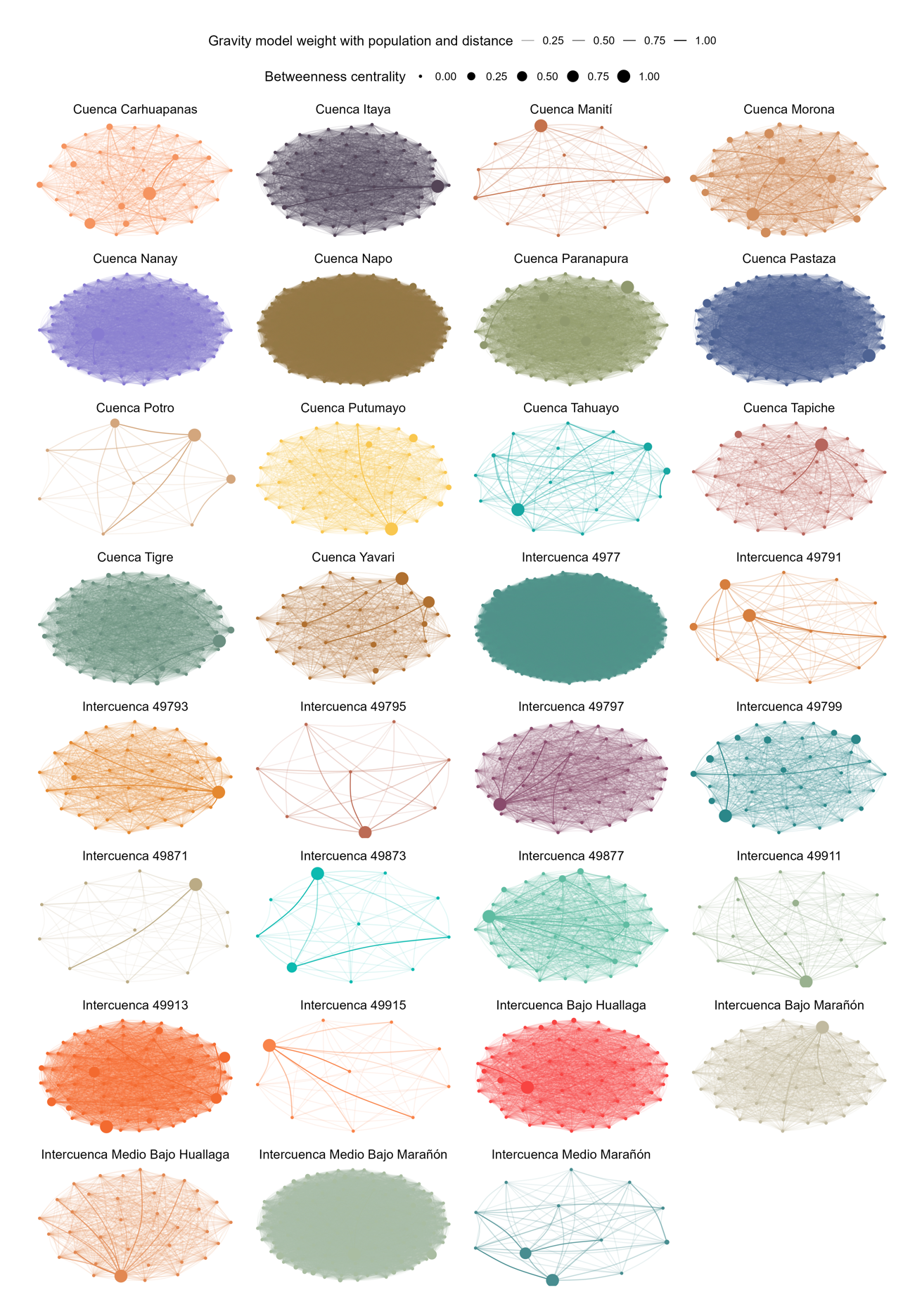
**
