# Supplementary material for: The role of connectivity on malaria dynamics across areas with contrasting control coverage in the Peruvian Amazon
Source: PLoS Negl Trop Dis. 2024 Nov 4;18(11):e0012560. doi: 10.1371/journal.pntd.0012560 (PMC11534198; doi:10.1371/journal.pntd.0012560)

**Supplementary Figure 5. Correlation of centrality metrics of villages in the Loreto department in the Peruvian Amazon.** Margin plots shows the mass (population [pop; light orange], deforested area [adef; light blue], and none [red]), cost (distance [d; green], travel time [t; purple]), and centrality type (betweenness [between; blue], strength [stre; orange], eigen [eigen; pink], closeness [close; light green]) used for the calculations. Dendrograms to cluster centrality metrics is based on a hierarchical cluster analysis using a complete linkage method.


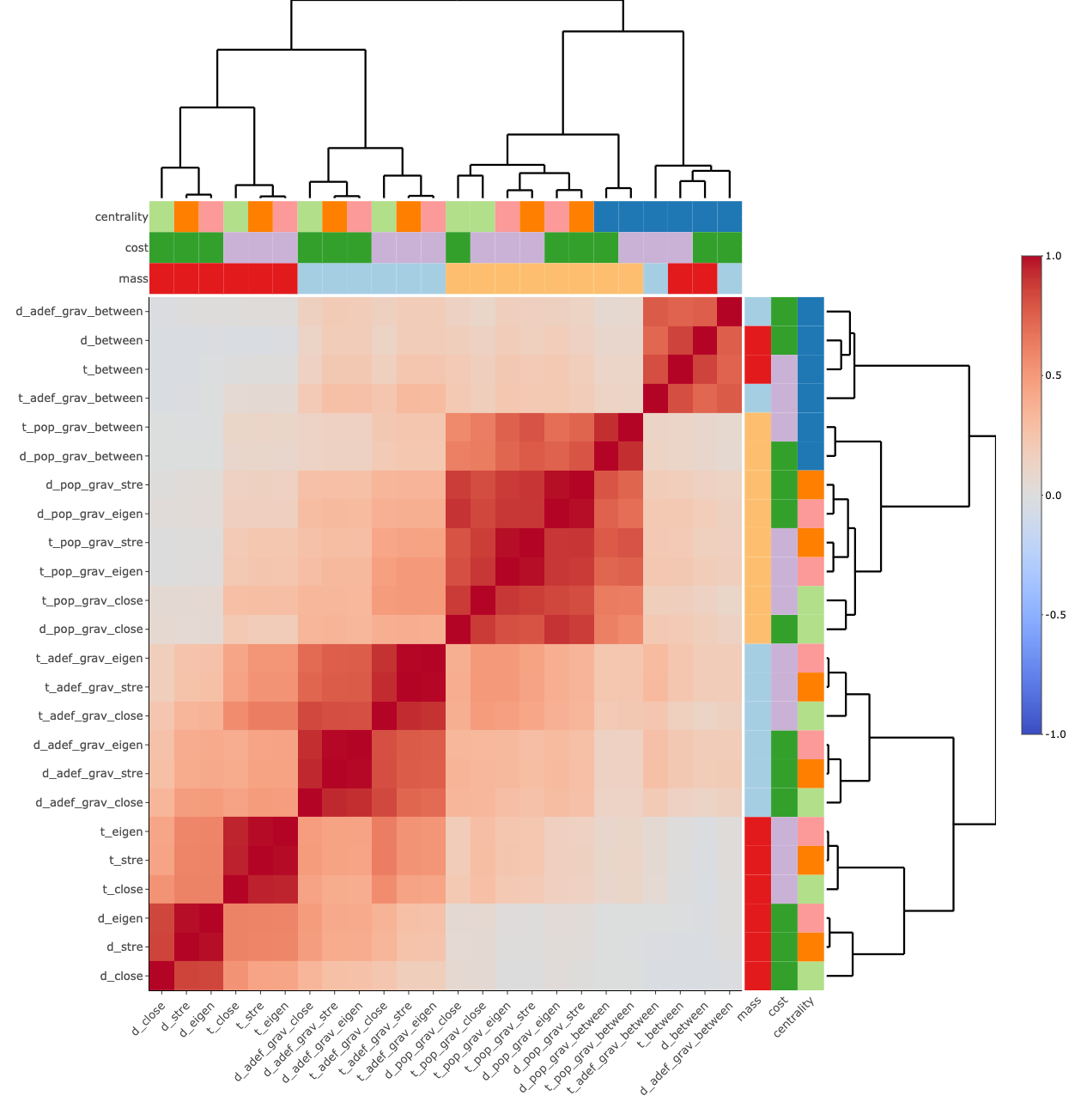

Supplement: S5 Fig — (DOCX) [file pntd.0012560.s008.docx]
