# Supplementary material for: The role of connectivity on malaria dynamics across areas with contrasting control coverage in the Peruvian Amazon
Source: PLoS Negl Trop Dis. 2024 Nov 4;18(11):e0012560. doi: 10.1371/journal.pntd.0012560 (PMC11534198; doi:10.1371/journal.pntd.0012560)

**Supplementary Figure 6. Distribution of Total Annual Parasite Index (API) per 1000 population (2011-2018) across high and low centrality villages stratified by levels of PAMAFRO intervention coverage and overall distributions in the Loreto department in the Peruvian Amazon.** A) For *Plasmodium vivax* and B) for *P. falciparum.* Mean difference between groups are represented as diamonds in each panel. T1 = Tier 1 (Low coverage; Low baseline endemicity), T2 = Tier 2 (Moderate coverage; Moderate baseline endemicity), and T3 = Tier 3 (High coverage; High baseline endemicity).

***A)***


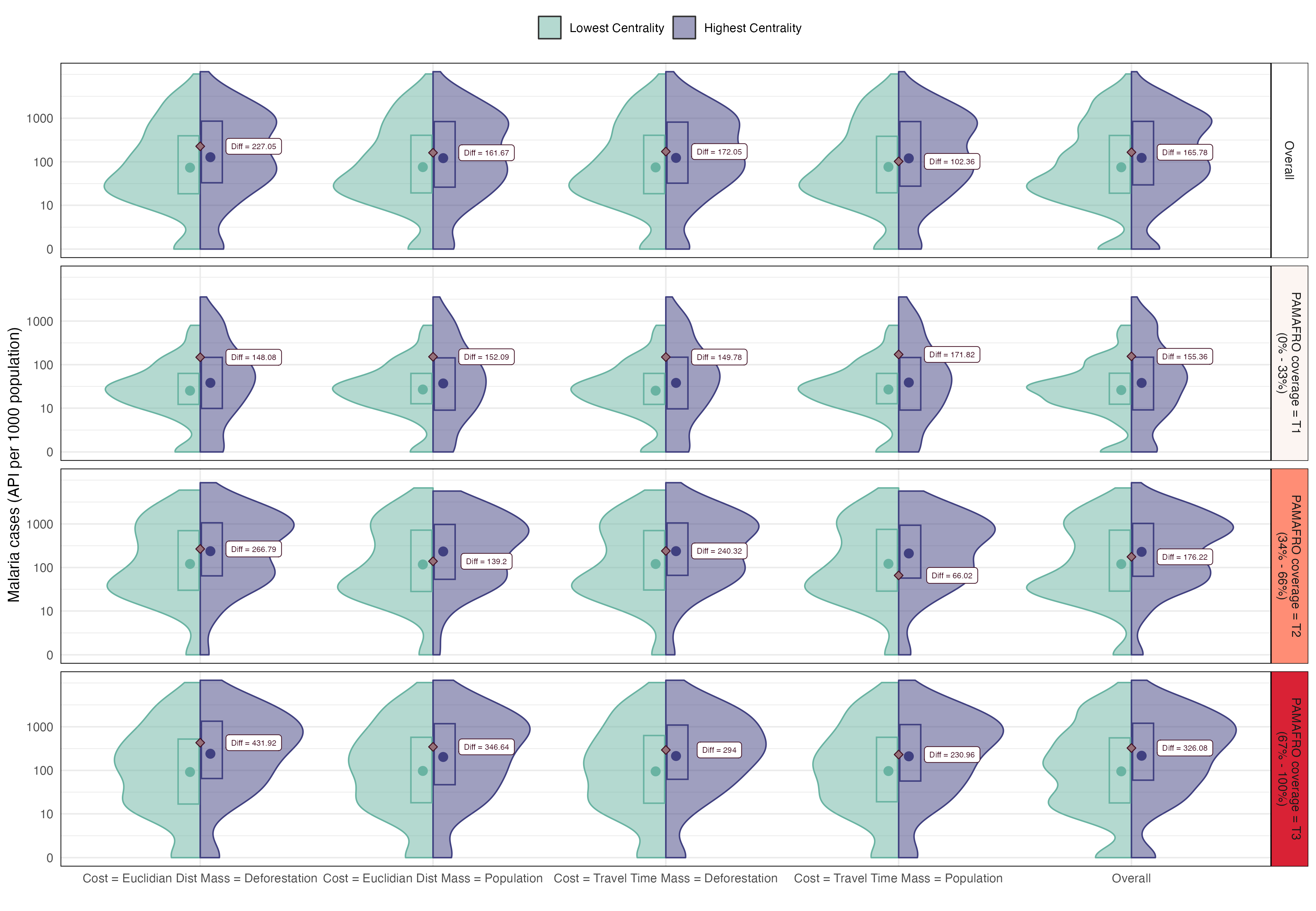


***B)***


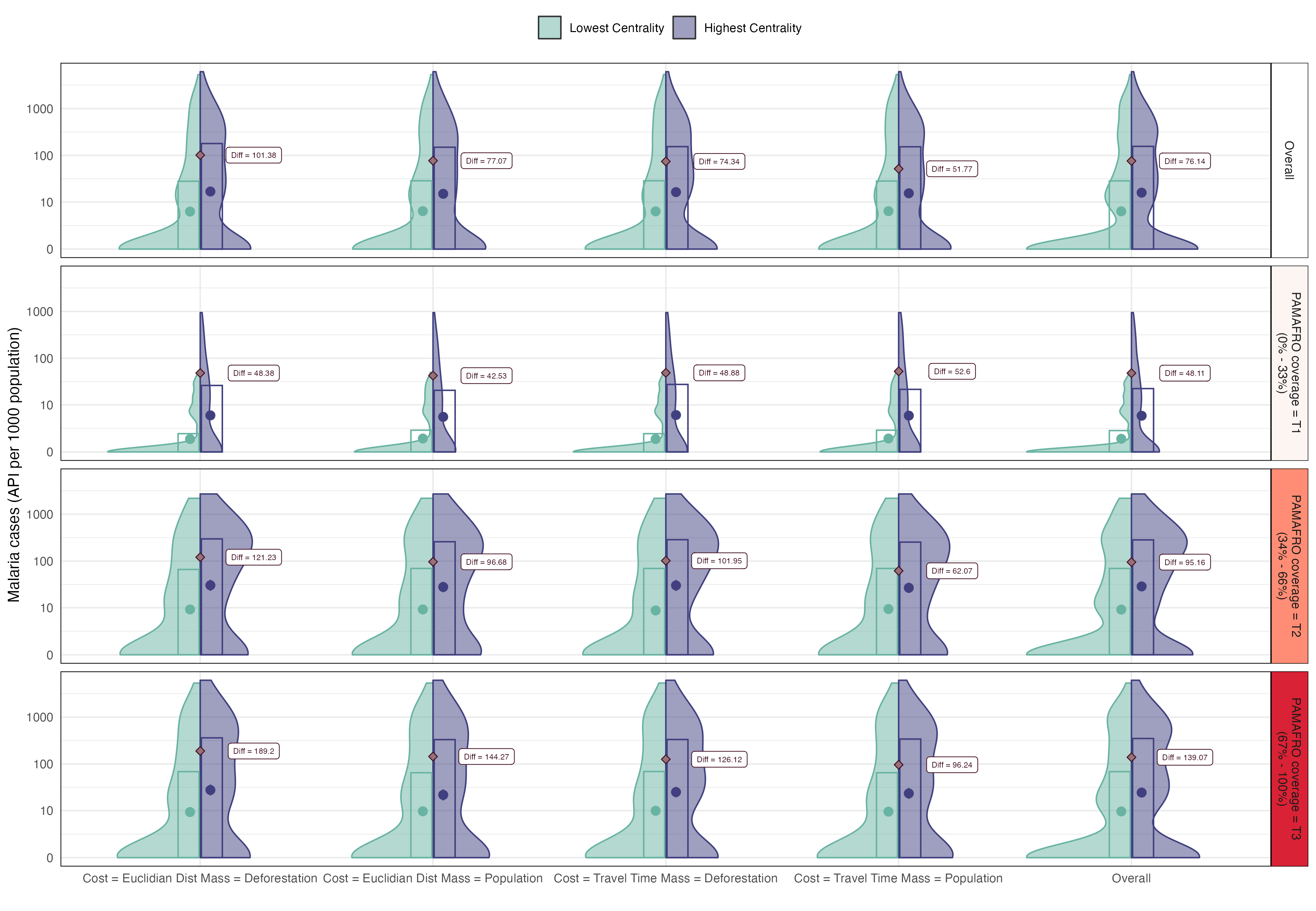

Supplement: S6 Fig — (DOCX) [file pntd.0012560.s009.docx]
