# Supplementary material for: The role of connectivity on malaria dynamics across areas with contrasting control coverage in the Peruvian Amazon
Source: PLoS Negl Trop Dis. 2024 Nov 4;18(11):e0012560. doi: 10.1371/journal.pntd.0012560 (PMC11534198; doi:10.1371/journal.pntd.0012560)
Supplement: Attachment — Submitted filename: CH02_Rebutal_letter.docx [file pntd.0012560.s010.docx]

UNIVERSITY OF CALIFORNIA, SAN DIEGO	UCSD

-------------------------------------------------------------------------------------------------------------------------------------------------------------------------------------------------------------------------------------------------------------------------------------------------------------------------------------------------------------------------------------------

BERKELEY	DAVIS	IRVINE	LOS ANGELES	RIVERSIDE	SAN DIEGO	SAN FRANCISCO	SANTA BARBARA SANTA CRUZ

------------------------------------------------------------------------------------------------------------------------------------------------------------------------------------------------------------------------

DEPARTMENT OF MEDICINE, DIVISION OF INFECTIOUS DISEASES	9500 GILMAN DRIVE

SCHOOL OF MEDICINE	LA JOLLA, CALIFORNIA 92093-0741

August 09, 2024

Dear Editor,

We were pleased to receive your invitation to revise and resubmit our manuscript for consideration and publication in PLOS Neglected Tropical Diseases, subject to adequate revision and response to the comments raised by the reviewers and editorial board members.

We would like to thank the reviewers for their thoughtful comments on our manuscript entitled "*The role of connectivity on malaria dynamics across areas with contrasting control coverage in the Peruvian Amazon* " (PNTD-D-24-00712). These comments have really improved the manuscript and contributed to the clarification of some critical points.

Based on the instructions provided, we have uploaded a copy of the original manuscript marked with all the changes made during the revision process and a clean version. Appended to this letter is our point-by-point response to the comments raised by the referees.

The manuscript has not been previously published and is not under consideration or substantially similar form in any other journal.

Sincerely,

Gabriel Carrasco-Escobar, MSc

Scripps Institution of Oceanography

University of California, San Diego

La Jolla, CA, USA

Email: [gacarras@health.ucsd.edu](mailto:gacarras@health.ucsd.edu)

**Reviewer #1:**

1. The results section doesn't directly address the research question (likely about the relationship between connectivity metrics and malaria incidence). While it describes various aspects of the data (e.g., baseline characteristics, centrality estimates), it doesn't show how these relate to malaria incidence. Start the results section by summarizing the main findings related to the research question and refocus the discussion on these findings.

> We have revised the Results section to directly address the research question by summarizing the main findings related to the relationship between connectivity metrics and malaria incidence with the following paragraph: “*The main findings of this study indicate a significant relationship between connectivity metrics and malaria incidence. High malaria incidence was linked to highly connected villages, especially near Iquitos and in deforested areas. Betweenness centrality consistently demonstrated the highest stability among 24 computed metrics, highlighting its importance in understanding malaria transmission. Villages in the top quintile of betweenness centrality had significantly higher malaria incidence compared to those in the bottom quintile, with this relationship being more pronounced in areas with higher PAMAFRO coverage. In the following section we will describe the baseline characteristics of the villages and centrality estimates to provide further context to our findings, demonstrating how centrality patterns are related to higher malaria rates*”.

Additionally, the Discussion section has been refocused to highlight how these findings inform our understanding of malaria transmission dynamics: “*Our findings indicate that betweenness centrality, among the various connectivity metrics, consistently and stably predicts malaria incidence. This result held true across different weighting methods, including population, deforested area, Euclidean distance, and travel time. Specifically, regions with higher betweenness centrality consistently exhibited higher malaria incidence rates. Further analysis revealed that this pattern is particularly pronounced in areas with extensive malaria control activities from the PAMAFRO project, which initially targeted regions with high baseline malaria transmission. This underscores the need to reconsider current malaria control strategies. Instead of focusing solely on locations based on their malaria metrics, our findings support a shift towards strategies that target connectivity neighborhoods, incorporating influential connected villages that facilitate the flow of parasites and hosts. Understanding how villages and cities are connected and how these connections influence the transmission of pathogens is of paramount importance for global public health. This study contributes to the limited literature on human mobility and its impact on malaria in rural areas of the Amazon region by investigating this relationship in the Peruvian Amazon. We utilized a comprehensive set of connectivity metrics and malaria incidence records at a granular spatial resolution. In summary, our study highlights the critical role of human connectivity in malaria transmission in the Loreto department of Peru. By prioritizing highly connected regions, we can enhance the effectiveness of malaria control efforts and move closer to the goal of malaria elimination*.”

2. What is the specific aim of investigating human population mobility and its connection to malaria transmission?

> We have clarified the specific aim of the study in the Introduction section, emphasizing the focus on understanding the influence of human population mobility on malaria transmission: “*In consequence, in this study we aimed to investigate how human population mobility, quantified through various connectivity metrics, influences malaria transmission by different levels of PAMAFRO coverage in the Loreto department of Peru between 2011 to 2018*.”

3. How will understanding the role of human mobility inform malaria control efforts and potentially lead to elimination?

> The Introduction section has been updated to explain how understanding human mobility can inform malaria control efforts and contribute to the goal of malaria elimination: “*Therefore, understanding the role of human mobility in malaria transmission is critical for informing control efforts. By identifying how mobility patterns contribute to the spread of malaria, we can develop more effective intervention strategies that target highly connected regions, ultimately aiding in the goal of malaria elimination*.”

4. While the formulas for Euclidean distance are provided, it would be helpful to explain any assumptions made or limitations of using Euclidean distance instead of actual road network distances.

> We have expanded the Methods section to explain the assumptions and limitations of using Euclidean distance instead of actual road network distances, providing context for the chosen approach. In addition, to have a more realistic representation we used time to travel. “*Euclidean distance assumes direct travel paths and may not accurately represent actual travel routes, which can be influenced by road networks and geographical barriers. Despite this limitation, Euclidean distance provides a simplified and computationally efficient method to analyze connectivity*."

5. The section cites employing speed restrictions for different land cover categories, however it does not explain how the accuracy of these estimates was verified.

> We appreciate your insightful comment regarding the verification of speed restriction estimates for different land cover categories. Indeed, we did not validate the accuracy of these metrics in our study, and we acknowledge this as a limitation. Due to logistical impediments, we were unable to conduct the necessary local-level validations. We recognize the importance of validating these estimates and suggest that future research should include this verification step to enhance the reliability of travel time estimations in similar studies. Thank you for pointing out this critical aspect: “*Accuracy of speed restriction estimates should be validated with mayor precision at local level in future studies to better represent study settings.*”

6. Are they any specific reasons why the authors used the six measure of centrality mentioned?

**>** The Methods section has been updated to explain the rationale for using the six specific centrality measures, detailing how each measure contributes to a comprehensive understanding of connectivity: “*These measures were chosen to provide a comprehensive view of how well-connected regions are within the network, each highlighting different facets of influence and accessibility. This metrics evaluated crucial aspects such as: 1) the number of direct connections to a node, providing insights into immediate contacts, 2) the average shortest path from a node to all others, reflecting accessibility and potential for rapid dissemination, 3) nodes that serve as bridges within the network, crucial for understanding pathways of pathogen transmission, and 4) the influence of a node's connections, highlighting nodes connected to other well-connected nodes*.”

7. The authors highlighted various limitations in the paragraph addressing the study limitations, but did not explain how these may affect the findings.

> We have elaborated on the impact of study limitations in the Discussion section, explaining how they may affect the findings and emphasizing the robustness of the observed relationships: “*We acknowledge some limitations of this study. First, 221 villages (12%) were excluded from the analysis in the data cleaning process due to missing data of the masses and costs for the weight calculations (Supplementary Figure 1). These exclusions may alter the estimations; however, data was missing completely at random (MCAR), reducing possibilities to overestimate or underestimate our findings. Second, for the connectivity metrics computation, all villages located in the same watershed were assumed to be connected. However, is plausible that human travel preferences might involve avoiding certain villages within the same watershed or traveling to villages in different watersheds. Future studies should consider more complex network structures to address these dynamics. While collapsing data for the entire study period (2011-2018) simplifies our understanding of the relative contribution of spatial units containing nodes/villages on malaria incidence, it is important to recognize the loss of temporal granularity. During epidemics, the centrality value of a node can fluctuate significantly, reflecting shifts in malaria transmission dynamics and being influenced by migration patterns and seasonal variations ^1^. Finally, previous studies in the Peruvian Amazon ^2-4^ reported a high number of sub-clinical infections that are not recorded by the MoH during routine data collection. The findings of this study are relevant only for clinical cases, and caution is suggested when interpreting these results for asymptomatic cases, which can contribute to the maintenance of parasite transmission. Therefore, incorporation of active-case surveillance data will be crucial to address this limitation in future studies*.”

**Reviewer #2:**

1. Figure 4. Define meaning of the colors.

> Figure 4 was changed by adding a legend explaining the meaning of the colors.

2. What was the long-term impact of PAMAFRO on malaria transmission in Loreto and which would be a sound control policy? This is an important question to address given the results presented.

> We have added a detailed discussion on the long-term impact of PAMAFRO on malaria transmission in Loreto: “*PAMAFRO intervention was temporally associated with a decrease in malaria transmission in Loreto Department ^5^. However, from 2012 to the present, both P. vivax and P. falciparum malaria cases have rapidly increased, highlighting the fragile nature of these gains ^5,6^. The interruption of PAMAFRO led to a resurgence of malaria in Loreto, with precipitation and actual evapotranspiration having a more significant effect on P. falciparum compared to P. vivax ^7^. This implicates that malaria control policies should include the re-establishment of continuous vector control measures, community health education, and robust surveillance systems ^8–10^. Therefore, Incorporating climate data into predictive models can help in anticipating and mitigating outbreaks ^6^*.”

3. Data were collapsed for the entire study period (2011-2018) and if this seems an appropriate way to simplify our understanding of the relative contribution of spatial units containing nodes/villages on malaria incidence, at the micro-epidemiological level information is lost on how the contribution of a node varies in time (e.g. during epidemics). In this sense, the centrality value for a node may change in time (e.g. Knudson et al, 2020; <https://doi.org/10.1038/s41598-020-60676-1>).

> We have now included a discussion on how collapsing data for the entire study period may obscure these temporal changes. We have cited Knudson et al. (2020) to illustrate how centrality values can fluctuate during epidemics and emphasized the need for future studies to consider these temporal variations for a more nuanced understanding of malaria transmission networks: “*While collapsing data for the entire study period (2011-2018) simplifies our understanding of the relative contribution of spatial units containing nodes/villages on malaria incidence, it is important to recognize the loss of temporal granularity. During epidemics, the centrality value of a node can fluctuate significantly, reflecting shifts in malaria transmission dynamics and being influenced by migration patterns and seasonal variations ^1^*.”

4. Which is the impact on malaria incidence of nodes with different centrality values in the network? How to evaluate directed graphs with different indegree and outdegree values for a node?

> We have expanded the discussion to include the impact of nodes with different centrality values on malaria incidence: “*Nodes with high degree centrality, particularly those with high indegree, are crucial importation points and potential outbreak hubs, while nodes with high outdegree are significant in exporting malaria to other areas. Evaluating directed graphs with differing indegree and outdegree values for each node allows us to identify and target these critical points within the network. Nodes with high centrality values were pivotal during malaria epidemics, indicating that these nodes play a significant role in the spread of the disease ^1^. In addition, Woolhouse et al.^11^ and Smith et al.^12^ emphasize the importance of tailored interventions based on the population biology of multihost pathogens and the basic reproductive number for malaria. This finding suggests that intensified surveillance and targeted control measures at these nodes could substantially reduce transmission rates*.”

**References:**

1	Knudson A, González-Casabianca F, Feged-Rivadeneira A, et al. Spatio-temporal dynamics of Plasmodium falciparum transmission within a spatial unit on the Colombian Pacific Coast. Sci Rep 2020; 10: 3756.

2	Carrasco-Escobar G, Miranda-Alban J, Fernandez-Miñope C, et al. High prevalence of very-low Plasmodium falciparum and Plasmodium vivax parasitaemia carriers in the Peruvian Amazon: insights into local and occupational mobility-related transmission. Malar J 2017; 16: 415.

3	Roshanravan B, Kari E, Gilman RH, et al. Endemic malaria in the Peruvian Amazon region of Iquitos. Am J Trop Med Hyg 2003; 69: 45–52.

4	Rovira-Vallbona E, Contreras-Mancilla JJ, Ramirez R, et al. Predominance of asymptomatic and sub-microscopic infections characterizes the Plasmodium gametocyte reservoir in the Peruvian Amazon. PLoS Negl Trop Dis 2017; 11: e0005674.

5	Rosas-Aguirre A, Gamboa D, Manrique P, et al. Epidemiology of Plasmodium vivax Malaria in Peru. Am J Trop Med Hyg 2016; 95: 133–44.

6	Janko MM, Recalde-Coronel GC, Damasceno CP, et al. The impact of sustained malaria control in the Loreto region of Peru: a retrospective, observational, spatially-varying interrupted time series analysis of the PAMAFRO program. Lancet Reg Health – Am 2023; 20. DOI:10.1016/j.lana.2023.100477.

7	Carrasco-Escobar G, Qquellon J, Villa D, Cava R, Llanos-Cuentas A, Benmarhnia T. Time-Varying Effects of Meteorological Variables on Malaria Epidemiology in the Context of Interrupted Control Efforts in the Amazon Rainforest, 2000-2017. Front Med 2021; 8: 721515.

8	Kaneko A. A community-directed strategy for sustainable malaria elimination on islands: Short-term MDA integrated with ITNs and robust surveillance. Acta Trop 2010; 114: 177–83.

9	Ng’ang’a PN, Aduogo P, Mutero CM. Strengthening community and stakeholder participation in the implementation of integrated vector management for malaria control in western Kenya: a case study. Malar J 2021; 20: 155.

10	Asale A, Kussa D, Girma M, Mbogo C, Mutero CM. Community based integrated vector management for malaria control: lessons from three years’ experience (2016–2018) in Botor-Tolay district, southwestern Ethiopia. BMC Public Health 2019; 19: 1318.

11	Woolhouse MEJ, Taylor LH, Haydon DT. Population Biology of Multihost Pathogens. Science 2001; 292: 1109–12.

12	Smith DL, McKenzie FE, Snow RW, Hay SI. Revisiting the Basic Reproductive Number for Malaria and Its Implications for Malaria Control. PLOS Biol 2007; 5: e42.
